# The Book World of Medicine and Science

**Published:** 1896-01-04

**Authors:** 


### THE BOOK WORLD OF MEDICINE AND SCIENCE.

A MANUAL OF BOTANY. By T. REYNOLDS GREEN, F.R.S., Sc.D., F.L.S., Professor of Botany to the Pharmaceutical Society of Great Britain, &c., &c. Vol. I. Morphology and Anatomy. (London: J. and A. Churchill.)

Professor Green's manual is intended to replace the older one by Bently, on which it is itself mainly based. It is well and clearly written, and will prove of service to those who wish to acquire a knowledge of the outlines of plant morphology. It is emphatically a text-book for students, the author having carefully avoided dealing with questions which are still under discussion, and this fact will probably cause disappointment to some who would have hoped for a more critical exposition from so well known an author. One or two points in the book however strike us somewhat unfavourably. Much confusion exists respecting the morphology of the inflorescence, and we cannot but think it a pity that Professor Green has not followed the extremely lucid and commonly used system of Eichler. In the diagrams of flowers also it would have been better had the position of the axis been marked, thus in Fig. 248, this should fall at the top, while in Fig. 250 it should fall to the side of the diagram. But notwithstanding a few blemishes, which are very difficult to avoid in books of this nature, the work is deserving of much praise as a whole, and not the least of its merits lies in the clearness and definitness, with which the subject matter is handled.

AIDS TO THE ANALYSIS OF FOOD AND DRUGS. By PERMAIN AND MOOR. (London: Baillière, Tindall, and Cox. 1895.)

The authors of this little aid have already shown by papers read before the Society of Public Analysts that they are fully competent to discuss the various methods at present in use for the detection and estimation of adulteration in foods and drugs. A perusal of the present volume discloses the fact that Messrs. Permain and Moor have maintained their reputation for thoroughness and exactness, as we find that the book, although of comparatively small dimensions, embraces succinct accounts of all the more important methods of food analysis. A few commercial articles foreign to the scope of the Sale of Food and Drugs Act, such as disinfectants, oils, and soaps, have been included in the present volume, and the information given affords the reader an opportunity of gaining an approximate knowledge of the means by which these materials are assayed and valued. In the latter half of the book a series of pharmaceutical drugs which are liable to adulteration are discussed, and the methods adopted for the determination of sophistication explained. This portion of the book might with advantage have been developed, as we do not believe that commercial integrity is such as to warrant the assumption that the many other drugs included in the Pharmacopœia are without reproach, so that the selection made by the authors is hardly likely to supply sufficient information to the medical man or public analyst in their endeavours to ascertain the purity of drugs submitted to them or required in their respective professions. As at the present time we are not acquainted with any handy volume in the English language which covers this particular ground, we are pleased to welcome its appearance, and congratulate the authors in accomplishing their work in so convenient and suitable form.

